# Cell phone based balance trainer

**DOI:** 10.1186/1743-0003-9-10

**Published:** 2012-02-08

**Authors:** Beom-Chan Lee, Jeonghee Kim, Shu Chen, Kathleen H Sienko

**Affiliations:** 1 Department of Mechanical Engineering, University of Michigan, Ann Arbor, USA; 2 Department of Electrical Engineering and Computer Science, University of Michigan, Ann Arbor, USA; 3 Institute of Gerontology, University of Michigan, Ann Arbor, USA; 4 Department of Biomedical Engineering, University of Michigan, Ann Arbor, USA

**Keywords:** vibrotactile, rehabilitation, sensory augmentation, balance, cell phone, smart phone, mobile phone

## Abstract

**Background:**

In their current laboratory-based form, existing vibrotactile sensory augmentation technologies that provide cues of body motion are impractical for home-based rehabilitation use due to their size, weight, complexity, calibration procedures, cost, and fragility.

**Methods:**

We have designed and developed a cell phone based vibrotactile feedback system for potential use in balance rehabilitation training in clinical and home environments. It comprises an iPhone with an embedded tri-axial linear accelerometer, custom software to estimate body tilt, a "tactor bud" accessory that plugs into the headphone jack to provide vibrotactile cues of body tilt, and a battery. Five young healthy subjects (24 ± 2.8 yrs, 3 females and 2 males) and four subjects with vestibular deficits (42.25 ± 13.5 yrs, 2 females and 2 males) participated in a proof-of-concept study to evaluate the effectiveness of the system. Healthy subjects used the system with eyes closed during Romberg, semi-tandem Romberg, and tandem Romberg stances. Subjects with vestibular deficits used the system with both eyes-open and eyes-closed conditions during semi-tandem Romberg stance. Vibrotactile feedback was provided when the subject exceeded either an anterior-posterior (A/P) or a medial-lateral (M/L) body tilt threshold. Subjects were instructed to move away from the vibration.

**Results:**

The system was capable of providing real-time vibrotactile cues that informed corrective postural responses. When feedback was available, both healthy subjects and those with vestibular deficits significantly reduced their A/P or M/L RMS sway (depending on the direction of feedback), had significantly smaller elliptical area fits to their sway trajectory, spent a significantly greater mean percentage time within the no feedback zone, and showed a significantly greater A/P or M/L mean power frequency.

**Conclusion:**

The results suggest that the real-time feedback provided by this system can be used to reduce body sway. Its advantages over more complex laboratory-based and commercial balance training systems in terms of cost, size, weight, functionality, flexibility, and accessibility make it a good candidate for further home-based balance training evaluation.

## Background

Postural imbalance can result from sensory abnormalities, infection, medications, aging, and various vestibular (central and peripheral), neurological, musculoskeletal, and vascular disorders [1,2]. Balance disorders increase the risk of non-fatal and fatal falls, leading to direct annual costs of approximately 19 billion USD [3]. Among the treatments available for balance disorders, balance rehabilitation has the advantage of being non-invasive while providing interventions that can be tailored to a patient's particular needs. These clinical balance rehabilitation programs are designed to recover, retrain, or develop new sensorimotor strategies, in order to facilitate functional mobility, decrease dizziness, and re-establish effective coordination [4-6]. Rehabilitation programs that incorporate motor, sensory, and cognitive systems are more effective than muscular training alone in reducing balance and coordination deficits [7-9].

Post-treatment, patients are instructed to continue exercises on their own at home, but lack of expert feedback has been shown to lead to reduced improvement, loss of motivation, and eventual discontinuation [7,10,11]. In addition, compliance decreases over time due to a lack of proper instruction (i.e., feedback on the appropriateness of exercise motions) and consequent loss of motivation [9]. Furthermore, practical considerations (e.g., costs, patient schedule, or therapist load) constrain the number of training sessions that can be performed in a clinical setting under expert supervision.

Sensory augmentation is a technique currently being explored as a means of supplementing compromised sensory information during rehabilitation in order to retrain sensorimotor function [12]. In the laboratory setting, balance has been improved with various real-time biofeedback display modalities including visual [13-16], auditory [17-20], electrotactile (applied to the tongue) [21-24], and/or vibrotactile [25-30]. The effects of surface electrode stimulation (i.e., galvanic vestibular stimulation) on postural performance have also been studied [31-34]. Existing biofeedback systems employing sophisticated inertial or center-of-pressure measurement devices [17,35], complex and high-resolution cue displays [24,27], or estimation algorithms for capturing body tilt [36] have been investigated for the purpose of task-oriented training in neuromotor rehabilitation [12].

Among these various biofeedback display modalities, vibrotactile feedback has the advantage of discreetly providing motion cues that may not interfere with a person's activities of daily living (e.g., hearing or speaking). Vibrotactile feedback displays can be co-located with the inertial measurement units (IMUs) used to detect the kinematics of a particular body segment, thereby providing more intuitive operation [37]. While positioning these components on the trunk offers less spatial resolution and increased reaction times compared to locations on the tongue, head, or finger [38], trunk positioning offers a significant advantage for rehabilitation purposes, since it maps directly to the body segment that primarily dictates the location of the center-of-mass with respect to the base-of-support. We have previously shown that real-time trunk-based vibrotactile feedback significantly decreases postural sway during multidirectional perturbed stances in individuals with vestibular deficits [27,39] and during normal and semi-tandem Romberg stances in older adults [40].

In their current laboratory-based form, vibrotactile feedback technologies are impractical for use in home rehabilitation training regimens due to their size, weight, complexity, calibration procedures, cost (due to high-performance IMUs and computational processors), and fragility. Very recently, commercial systems dedicated to balance training have become available [41,42]. The BalanceFreedom™ [41] measures angular deviations and angular velocities of the trunk and provides auditory, vibrotactile, and visual cues through a headband. The VertiGuard^® ^RT [42] is a vibrotactile feedback system that measures body sway and provides vibrotactile cues on the trunk with an intensity proportional to the magnitude of body sway in the direction of a vibrating actuator (tactor).

Both the BalanceFreedom™ and VertiGuard^® ^RT systems were developed in order to improve patients' balance stability during stance and gait in the clinical environment. However, wider use of vibrotactile feedback for balance rehabilitation can be achieved by taking advantage of technologies that are already widespread. Smartphones have particular advantages for this purpose, as they feature increasingly powerful microprocessors, considerable memory capacity, large screens, open source operating systems, tri-axial accelerometers, and high-resolution video, making them ideal candidates for easily programmable and customizable feedback of body motion. Supplying vibrotactile balance cues through a smartphone obviates the need to purchase and carry a dedicated system for those within the rapidly-expanding smartphone market, which is projected to reach 1 billion users by 2014 [43]. Furthermore, smartphones offer features that dedicated vibrotactile feedback systems do not, such as the ability to wirelessly communicate with a hospital or therapist through an internet data connection or Bluetooth, the support of a large programming community, and the ability to integrate balance training into a larger suite of smartphone-based medical applications that include real-time monitoring of physiological signals such as blood pressure [44], body temperature [45], and heart rate [46,47].

Recognizing these advantages related to increased functionality and improved access for at-home physical rehabilitation, we have designed, developed, and assessed a low-cost, small, lightweight, easy-to-use, smartphone-based vibrotactile feedback system for balance rehabilitation training. Our eventual goal is to develop an effective system that can be used in the home to assist a patient with therapist-assigned balance exercises or in an environment where access to balance therapy is limited (e.g., rural regions in the developing world, where health care access is difficult but cell phone networks are increasingly prevalent [48]). In what follows, we 1) describe the hardware and software design, 2) quantitatively assess the effectiveness of the proposed system in young healthy subjects and subjects with vestibular deficits, and 3) discuss the potential applications of this technology for clinical and home-based balance rehabilitation training. Preliminary reports pertaining to this study were published in abstract form [49,50].

## Methods

### Hardware design

**Figure 1:**
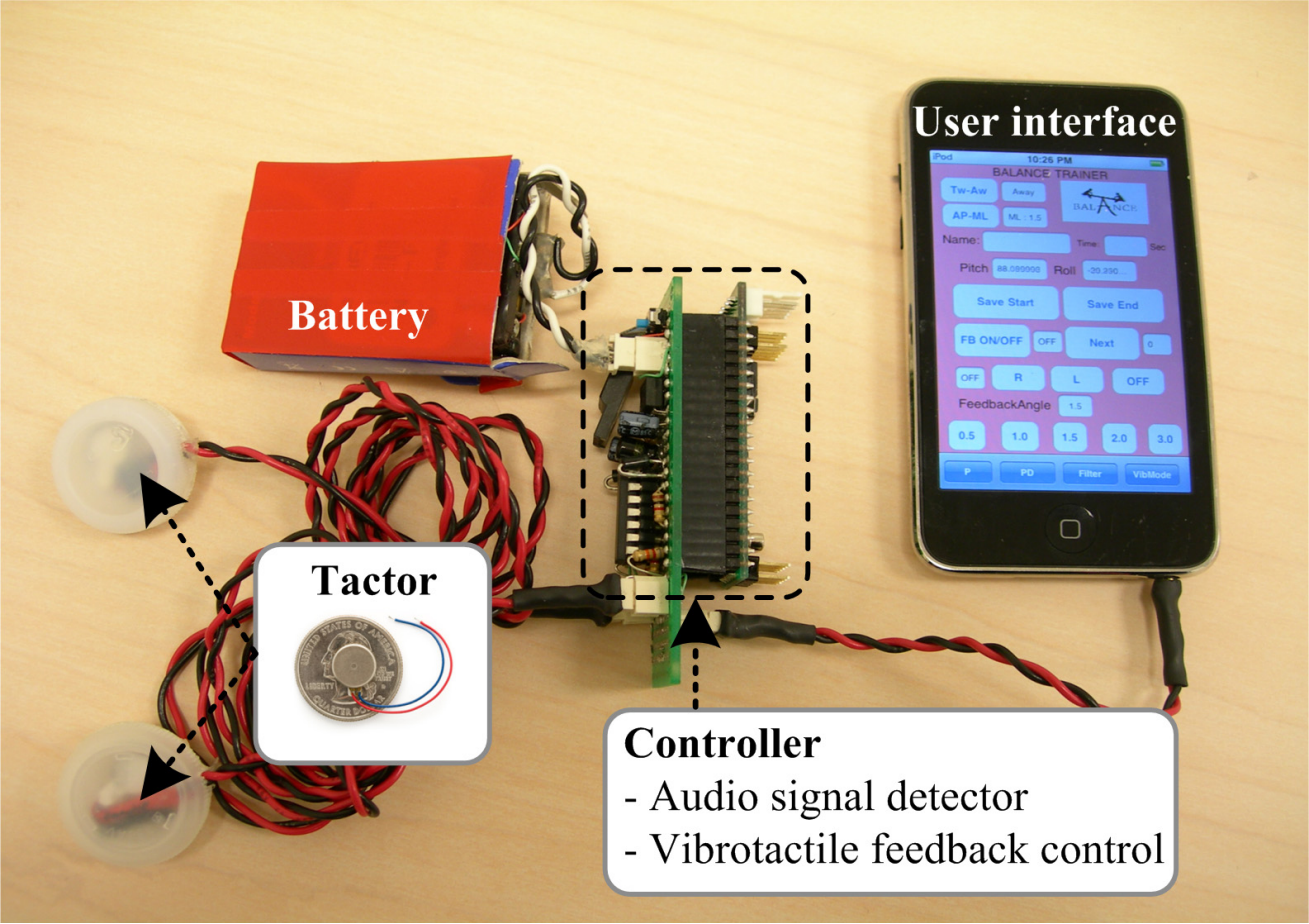
**Cell phone based balance trainer prototype**.

In order to provide vibrotactile feedback, we developed a hardware accessory referred to as a "tactor bud" which plugs into and receives sinusoidal signals from the cell phone audio jack. The tactor bud consists of a controller, battery, and two tactors as shown in Figure 1. Note that the tactor bud is much smaller and much lower in cost than the dedicated vibrotactile systems discussed above. The controller is composed of a microcontroller unit (MCU) (ATMEL, ATmega 32), quad operational amplifier (MC33204), and two band-pass filters with frequency ranges of 100-600 Hz and 1.9-3.0 kHz. It detects the frequency of the sine wave generated by the cell phone (either 250 Hz or 2 kHz) and provides a 3.0 V DC voltage signal to drive one of the two tactors based on which frequency is detected. All controller functions are managed by the MCU, which provides acceptable computational performance (each command is executed at a frequency of 16 MHz) with minimal power consumption at a low cost (less than $20/MCU).

The choice of tactor was based on a number of design considerations including availability, size, weight, power consumption, cost, and signal strength as perceived by the user. While we have previously demonstrated balance improvements in healthy subjects, older adults, and patients with vestibular deficits using laboratory-based vibrotactile feedback systems that employ C2 and Tactaid tactors [27,39,40,51], the high power consumption and cost of these tactors make them less suited for mobile battery-powered applications. Consequently, we chose small, low-power, coin-style tactors (Samsung Electro-Mechanics, DMJBRK30O, $4 each) that are actuated via eccentric mass pager motors which vary their rotation speed as a function of the DC voltage input. These round tactors each have a diameter of 10 mm, weight of 1.2 g, operation range of 2.5 V to 3.5 V at 65 mA, vibration frequency of 200 Hz at 3.0 V, and spin-up time of approximately 90 ms to reach maximum rotational velocity. Since they can be actuated by DC voltage alone, no complex controllers are required for signal generation. In order to create a larger skin contact area, we used 25 mm diameter plastic caps to house each tactor.

### Cell phone platform and software algorithms

For our initial design we sought to develop a cell phone platform that provided real-time operation, simple setup and use by the programmer and end user, integrated motion sensing, and the ability to save training data for subsequent performance analysis. We chose the iPhone (Apple, Inc. iPhone 3GS) for this purpose, as it includes a built-in tri-axial linear accelerometer (STMicoelectronics, LIS302DL), an adequately powerful microprocessor (ARM Cortex A8, 600 MHz), substantial memory capacity (16 GB storage), a touch screen interface (8.89 cm with 320 × 480 pixels), and a software development toolkit (SDK) provided by Apple, Inc. [52]. Mounted on the waist via an elastic belt, the phone was used to measure body acceleration, estimate both anterior-posterior (A/P) and medial-lateral (M/L) body tilt, provide tactor activation commands to the "tactor bud" vibrotactile stimulation hardware, and store body tilt data for later performance analysis.

Tilt estimates were computed using an Euler-angle-based extended Kalman filter (EKF) [53] with four state variables. Two of the state variables, angular positions in the pitch (*θ_pitch_*) and roll (*θ_roll_*) directions, were calculated from the output of the tri-axial accelerometer [54]:

(1) $$\begin{array}{c} \theta_{pitch}=-{\text{sin}}^{-1}\left(\frac{g_{x}}{{\left\|g\right\|}_{2}}\right)\\ \theta_{roll}={\text{sin}}^{-1}\left(\frac{g_{y}}{{\left\|g\right\|}_{2}\text{cos}\left(\theta_{pitch}\right)}\right) \end{array}$$

where ||*g*||_2 _is the magnitude of gravity (*g *= [*g_x _g_y _g_z_*]) and the subscript "2" indicates the Euclidean norm. The remaining two state variables (roll and pitch angular velocities) were calculated from time derivatives of the roll and pitch angular positions. The system and measurement models used within the EKF are expressed by

(2) $$\left\{\begin{array}{c} x_{k+1}=F_{k}x_{k}+w_{k}\\ y_{k}=H_{k}x_{k}+v_{k} \end{array}\right)$$

where **x_k+1 _**and **x_k _**denote the state vector at times **k+1 **and **k **and **y_k _**denotes the measurement vector at time **k**. **F_k _**and **H_k _**denote the coefficients at time **k **which determine the characteristics of the system model and measurement model, respectively. **W_k _**and **V_k _**denote system noise and measurement noise, respectively, at time **k**. Based on a first-order linear state transition model and a non-linear measurement model, the system model can be expressed as follows in order to determine body motion:

(3) $$\left[\begin{array}{c} x_{k+1}\\ {\dot{x}}_{k+1} \end{array}\right]=\left[\begin{array}{cc} 1 & \Delta \text{t}\\ 0 & 1 \end{array}\right]\;\;\left[\begin{array}{c} x_{k}\\ {\dot{x}}_{k} \end{array}\right]+\left[\begin{array}{c} w_{k,1}\\ w_{k,2} \end{array}\right]$$

where **W_k, 1 _**and **W_k, 2 _**are the system noise in angular position and angular velocity, respectively, at time **k**.

**Figure 2:**
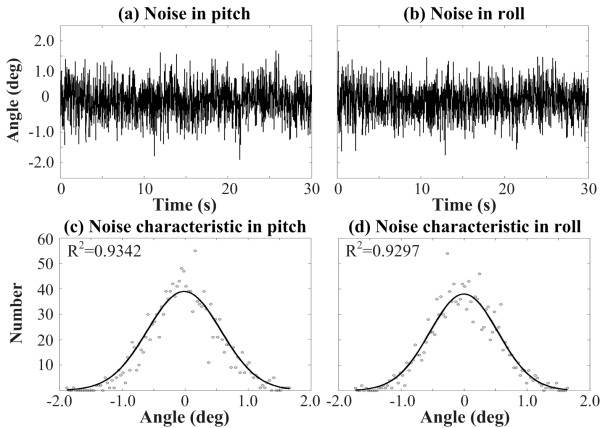
**Noise characteristics of angular positions in pitch and roll directions**. (a) Noise in pitch direction without the EKF estimation. (b) Noise in roll direction without the EKF estimation. (c) Predicted Gaussian curve (solid line) of noise distribution (points) in pitch direction. (d) Predicted Gaussian curve (solid line) of noise distribution (points) in roll direction. Note that values for R^2 ^range from 0 to 1 and indicate the goodness of fit between the predicted Gaussian curve and the noise distribution.

The EKF applies the linear Kalman filter to nonlinear systems with additive white noise (i.e., measurement noise) by iterating the state estimate after an initial guess [55]. In order to quantify measurement noise and drift, the phone (containing the tri-axial linear accelerometer sensor) was characterized on a vibration isolation (optical) table. Analysis of resulting measurement noise in pitch and roll angular positions showed a distribution resembling white Gaussian noise (Figure 2), supporting the use of a Kalman filter.

**Figure 3:**
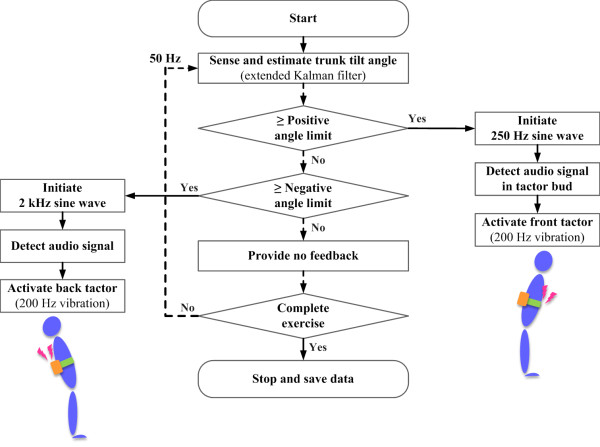
**Software architecture flow chart**.

Figure 3 shows a flow chart of the software architecture implemented in the cell phone platform. If the body tilt angle estimated by the EKF surpassed a preset positive angle limit with respect to the vertical, a 250 Hz sine wave was transmitted to the tactor bud through the iPhone's audio output jack. Similarly, if the estimated body tilt angle was less than the negative angle limit with respect to the vertical, a 2 kHz sine wave was transmitted. The generated sine waves were detected by the audio signal detector in the tactor bud hardware controller, which activated the proper tactor based on the sine wave frequency. The update rate from sensing to displaying vibrotactile feedback was nominally 50 Hz.

### Subjects

Five young, healthy, naïve subjects (24 ± 2.8 yrs; 3 females, 2 males) and four subjects with vestibular deficits (42.2 ± 13.5 yrs; 2 females, 2 males) participated in this proof-of-concept study. Three of the latter group were patients with unilateral vestibular deficits, and one had bilateral vestibulopathy. Patients were eligible to participate in the study if they had a diagnosed peripheral vestibular impairment, caloric weakness of 25% or greater on either side, and a recommendation by a physical therapist for balance rehabilitation. Subjects with vestibular involvement were excluded if they had severe visual impairment, history of fainting, idiopathic vestibulopathies, or neurological disease affecting balance (e.g., Parkinson's). The University of Michigan Institutional Review Boards approved the experimental protocol, which conformed to the Helsinki Declaration. Informed consent was obtained from each subject prior to the start of the experiment.

### Protocol

Both subject groups were instrumented with the cell phone based balance trainer. Healthy subjects were tested using three different stance conditions: Romberg, semi-tandem Romberg, and tandem Romberg stance. For the Romberg stance condition, tactors were placed on the trunk midline (navel and spine) at approximately the L4/L5 vertebrae level. For the semi-tandem Romberg and tandem Romberg stance conditions, tactors were placed on the medial and lateral sides of the trunk at approximately the L4/L5 vertebrae level. Both subject groups were instructed to move away from the vibrotactile cue until the vibration stopped [26-28,39]. The positive and negative angle limits (i.e., dead zone) beyond which vibrotactile feedback was activated were selected as ± 1.0° in the A/P direction for Romberg stance, ± 1.0° in the M/L direction for semi-tandem Romberg stance, and ± 1.5° in the M/L direction for Romberg stance.

Each subject performed practice trials for each stance condition before the experimental protocol began. Each practice trial consisted of a 40 s balance task followed by a 20 s break. Healthy subjects performed 12 practice trials (~15 min. total) with their eyes closed for each stance condition, including 10 trials with vibrotactile feedback and two trials without vibrotactile feedback. Subjects with vestibular deficits participated in a separate study involving a lab-based vibrotactile feedback system immediately before completing the cell phone study and consequently were well acquainted with the challenging nature of the semi-tandem Romberg stance both in the presence and in the absence of vibrotactile feedback.

For both subject groups, the experimental protocol comprised eight separate trials consisting of two trials without vibrotactile feedback, followed by four trials with vibrotactile feedback, followed by two trials without vibrotactile feedback. Healthy subjects were instructed to keep their eyes closed with arms crossed over their chest during all three stance conditions. The subjects with vestibular deficits were tested using only the semi-tandem Romberg stance, with their arms crossed over their chest during both the eyes-open and eyes-closed conditions, since the tandem Romberg stance proved too challenging to perform. The study team assisted all subjects with both donning and operating the cell phone system.

Following the completion of the experimental protocol, each subject answered a six-question comparative Likert scale survey (strongly disagree (1), disagree (2), neither disagree nor agree (3), agree (4), or strongly agree (5)) which assessed his/her preference for the device, balance confidence, and impression of the system's intuitiveness. The survey questions were: Q1) My body was more stable when feedback was available than when it was not available, Q2) The feedback did not distract me from performing the given balance task, Q3) When the feedback was available, I felt more confident in my ability to maintain my balance during the given balance task, Q4) I could use this type of feedback at home by myself if I were given the appropriate equipment, Q5) I understood how to use the feedback, and Q6) I would prefer to use no feedback rather than use this type of feedback.

### Data Analysis

All data post-processing was performed using MATLAB (The MathWorks, Natick, MA). The metrics used to quantify subjects' balance performance were root mean square (RMS) of body tilt, elliptical area (EA) of body sway trajectory, percent time spent in the dead zone (in which tactors are not activated) (PZ), and mean power frequency (MPF) of body tilt, calculated for each trial from the power spectral density of body tilt. RMS body tilt values in the A/P and M/L directions were calculated by computing the square root of the time average of the squared tilt values in the respective direction. The body tilt trajectory of each trial was fit with a 95% confidence interval ellipse in order to capture the sway area. In addition, PZ analysis was conducted by calculating the percentage of time that the body tilt was within the specified angle limits for a given stance condition. The MPF parameters of A/P and M/L tilt were computed to characterize the mean spectral decompositions of sway motions in specific bandwidths (0-1.0 Hz). Non-feedback and feedback trials were separately averaged for each subject for each metric.

Statistical analysis was performed using linear mixed effects models (LMM). One particularly desirable feature of this analytical methodology is that it takes into account the likely correlation of repeated measurements performed on the same subject. Dependent variables were RMS body tilt, EA, PZ, and MPF. The primary focus of the analysis was to estimate the effects of vibrotactile feedback during different stance conditions on the dependent variables while accounting for the correlation of the replicated measures obtained from the same subject. Hypotheses for the main effects of vibrotactile feedback were tested using an F-test. The averages of the dependent variables during the first two trials (which were performed without feedback) were used as baseline values in order to evaluate the effects of vibrotactile feedback and facilitate comparisons among subjects. Significance was defined at the *p *≤ 0.05 level.

The rank of vibrotactile feedback for all six survey questions was averaged over all subjects to determine an overall rank of the proposed system efficacy, with five being the highest rank and the 'most preferred' or 'most helpful in maintaining balance'.

## Results

### Performance evaluation of cell phone based vibrotactile balance training

**Figure 4:**
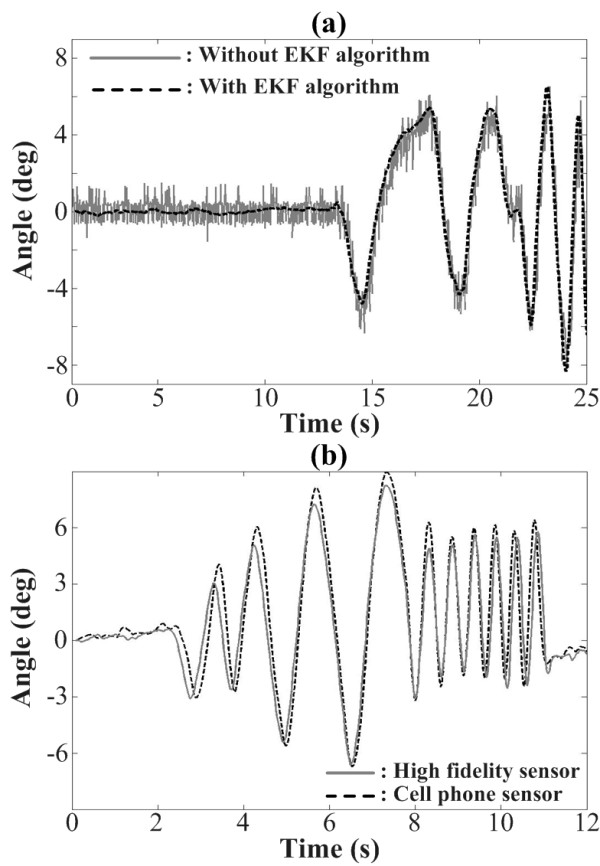
**(a) Effect of EKF algorithm on tilt estimation performance: grey solid and black dashed lines represent estimated tilt angle of the cell phone without or with EKF algorithm, respectively**. (b) Benchmarking of tilt estimation: grey solid and black dashed lines represent tilt angle estimated by a high fidelity sensor and the cell phone sensor, respectively. Tilt angle was sampled at a rate of 50 Hz for both sensors.

Figure 4 (a) shows the performance of the EKF tilt estimation algorithm implemented in the cell phone system. To evaluate accelerometer noise and drift, the cell phone was attached to a tilt table that was manually manipulated. Without the EKF algorithm, the noise of the angular position accelerometer output computed by Equation 1 was observed to be approximately ± 1.2°, while employment of the EKF algorithm reduced the noise to 0.2° in both pitch (A/P) and roll (M/L) directions without significant estimation delay.

To benchmark the precision of the cell phone accelerometer and tilt estimation algorithm, the computed tilt was compared with that of a high fidelity motion tracking IMU (Xsens, Xsens Technologies B.V., Enschede, NL.), as shown in Figure 4 (b). Both the cell phone and Xsens IMU were attached to a tilt table that was manually manipulated. At a tilt frequency of 2.5 Hz, the sensing resolution of the cell phone based system was determined to be better than 0.2°, while the high fidelity Xsens IMU achieved resolution better than 0.1°. Note that the data from the cell phone were recorded in the phone's internal memory while the data from the Xsens IMU were recorded on a laptop computer. While the rate for sampling, processing, and recording was set at 50 Hz for both systems, the systems exhibited variations (as measured by the CPU clocks) of ± 3.2 ms (± 16%) and ± 0.5 ms (± 2.5%) for the iPhone and laptop computer, respectively, leading to the slight synchronization discrepancy observed between the two signals in Figure 4(b).

### Effects of cell phone based vibrotactile balance training in healthy subjects

**Figure 5:**
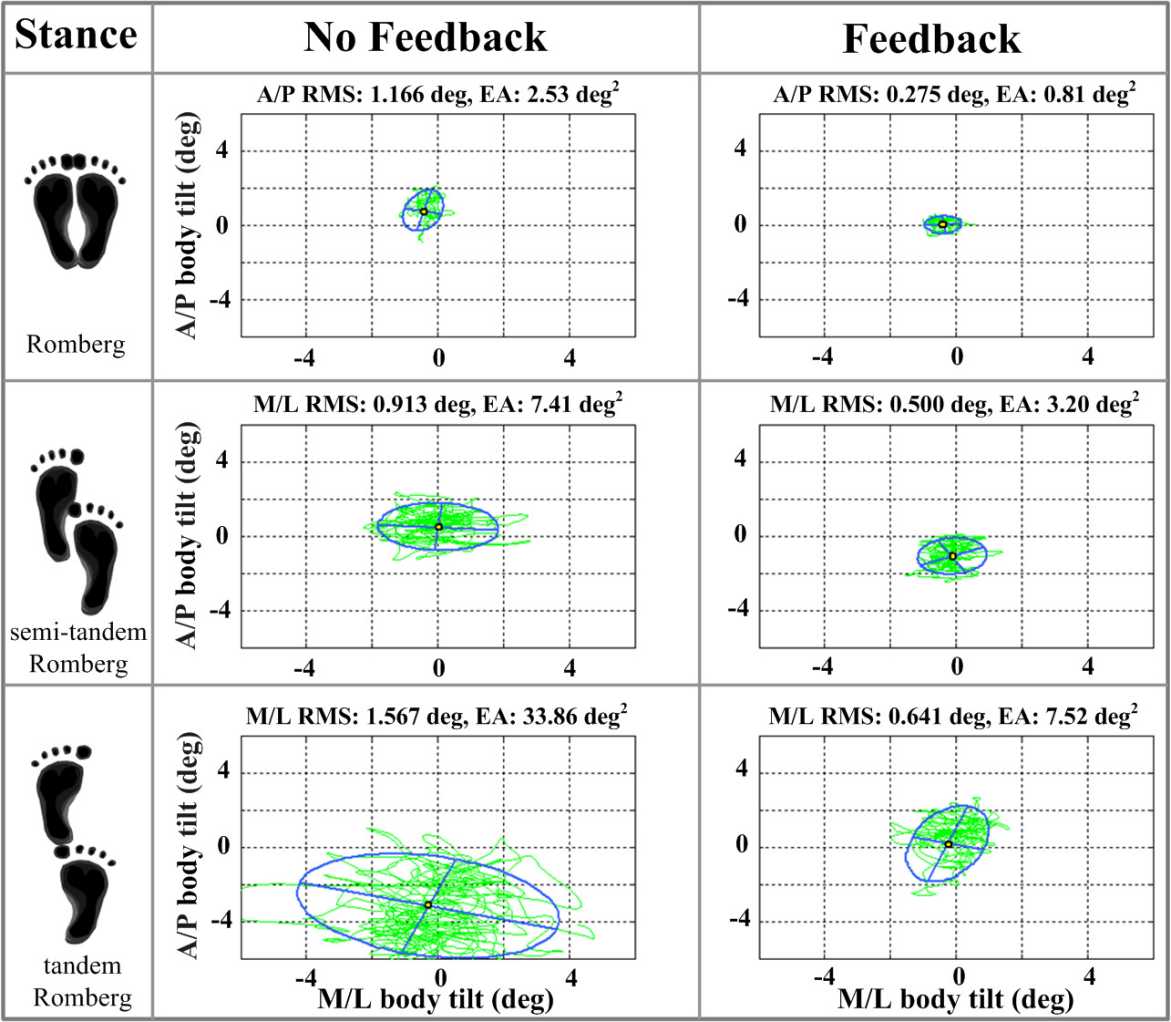
**Elliptical fits and RMS values for one healthy subject trial under each stance condition without (left column) and with (right column) feedback**. The blue line represents the elliptical fit with major and minor axes, and the green line represents the body tilt trajectory.

Figure 5 shows elliptical fits and RMS tilt values for example body sway trajectories obtained from a healthy subject for the three different stance conditions. The subject exhibited a decreased EA for each stance condition when cell phone based vibrotactile feedback was applied, as well as decreased RMS tilt. All healthy subjects demonstrated larger EA during tandem Romberg stance than during the other two stances, presumably due to the more challenging nature of the tandem Romberg stance.

**Figure 6:**
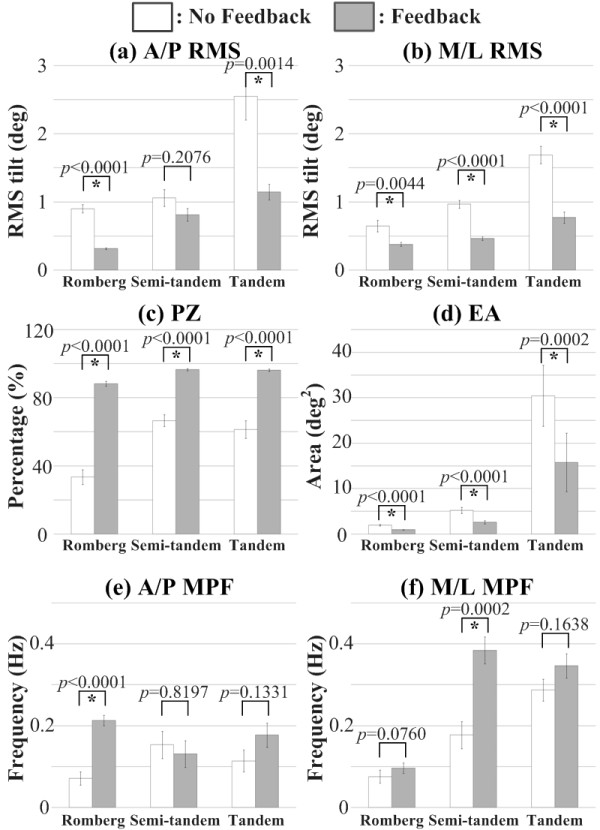
**Balance metric results for healthy subjects**. Error bars indicate standard error of the mean.

Figure 6 presents the statistical analysis and significance levels of the average A/P RMS, M/L RMS, PZ, EA, A/P MPF, and M/L MPF metrics for healthy subjects during all stance conditions. For Romberg stance, significant main effects were found for the A/P RMS, M/L RMS, PZ, EA, and A/P MPF metrics when vibrotactile feedback was provided. Subjects showed a significant decrease in A/P RMS, M/L RMS, and EA, and a significant increase in PZ and A/P MPF. For semi-tandem Romberg stance, significant main effects were found for the M/L RMS, PZ, EA, and M/L MPF metrics when vibrotactile feedback was provided. Subjects showed a significant decrease in M/L RMS and EA, and a significant increase in PZ and M/L MPF. For tandem Romberg stance, significant main effects were found for the A/P RMS, M/L RMS, PZ, and EA metrics when vibrotactile feedback was provided. Subjects showed a significant decrease in A/P RMS, M/L RMS, and EA, and a significant increase in PZ.

Frequency domain analysis of body tilt (MPF) for healthy subjects showed that provision of vibrotactile feedback significantly increased low frequency power in the 0.1 Hz to 0.4 Hz range for both the A/P direction during Romberg stance and the M/L direction during semi-tandem Romberg stance.

### Effects of cell phone based vibrotactile balance training in subjects with vestibular deficits

**Figure 7:**
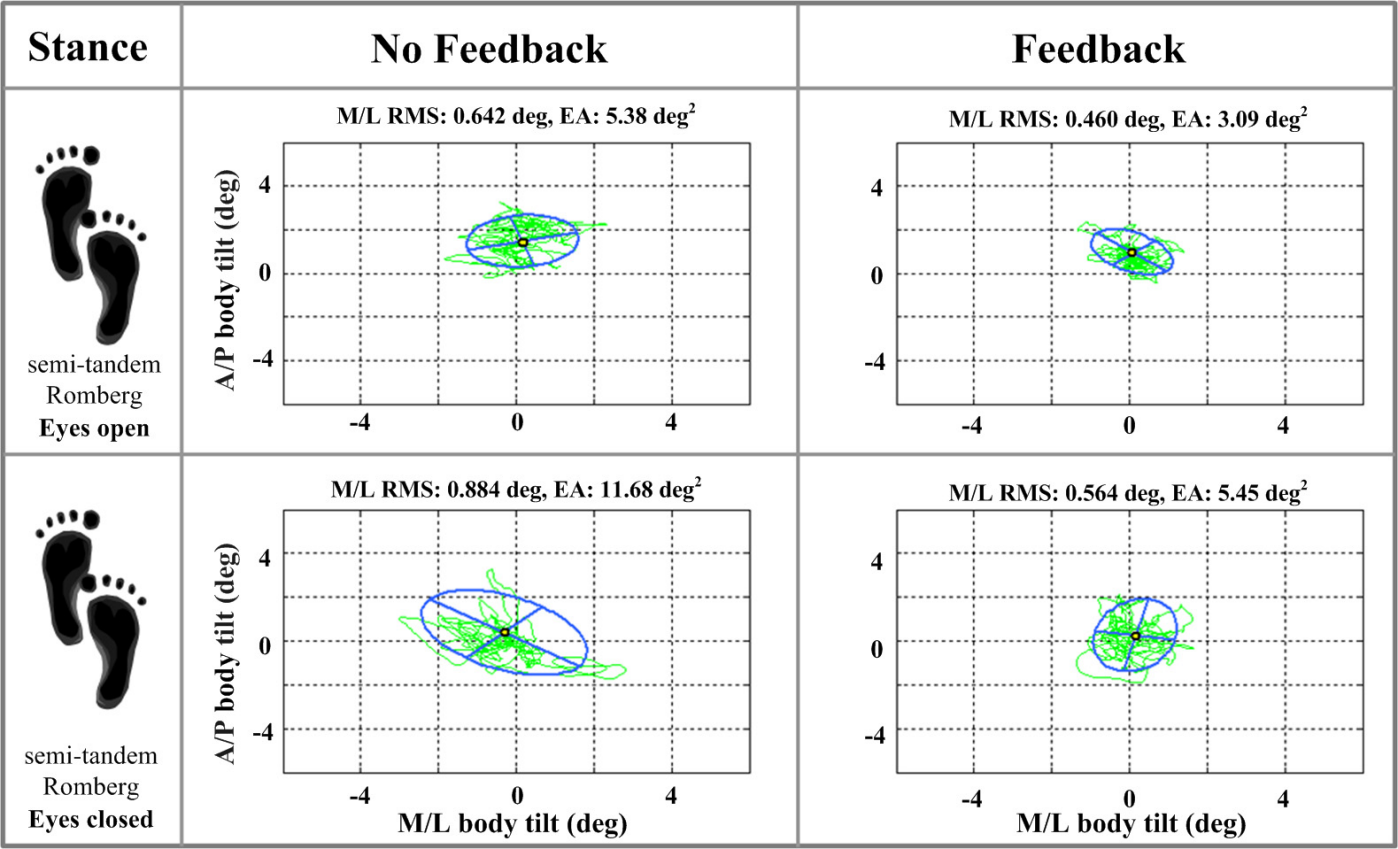
**Elliptical fits and RMS values for one subject with a vestibular deficit during eyes-open and eyes-closed conditions**. Left and right columns indicate the subject's performance without and with feedback, respectively. The blue line represents the elliptical fit with major and minor axes, and the green line represents the body tilt trajectory.

Figure 7 shows elliptical fits and RMS tilt values for example body sway trajectories obtained from a subject with vestibular loss during eyes-open and eyes-closed testing in the semi-tandem Romberg stance. While an increase in EA was observed for the eyes-closed condition relative to the eyes-open condition for both feedback conditions, the subject exhibited a decrease in EA and RMS for both eyes-open and eyes-closed conditions when cell phone based vibrotactile feedback was applied.

**Figure 8:**
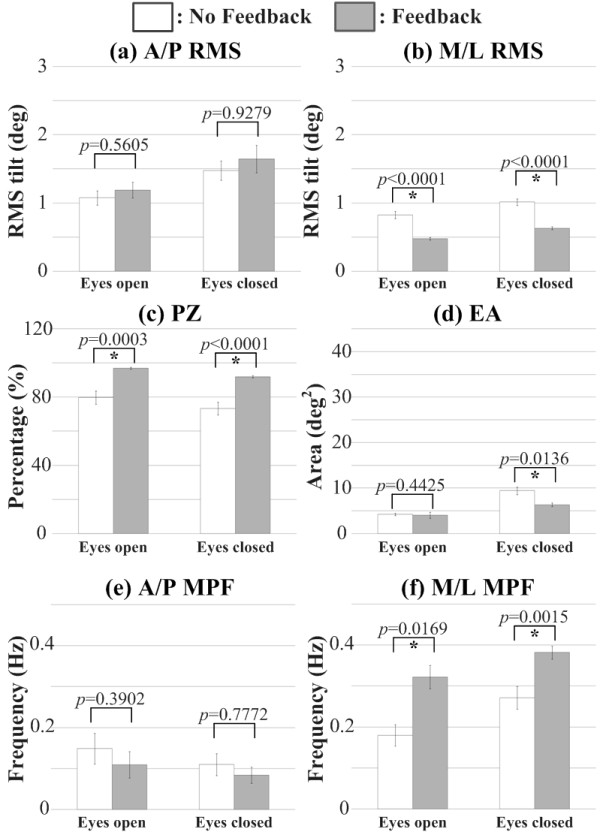
**Balance metric results for subjects with vestibular involvement**. Error bars indicate standard error of the mean.

Figure 8 presents the statistical analysis and significance levels of the A/P RMS, M/L RMS, PZ, EA, A/P MPF, and M/L MPF metrics during the semi-tandem Romberg stance condition for subjects with vestibular deficits. Significant main effects were found for the M/L RMS, PZ, EA, and M/L MPF metrics when vibrotactile feedback was provided, regardless of whether subjects had their eyes open or closed. Subjects showed a significant decrease in M/L RMS and EA, and a significant increase in PZ and M/L MPF. When subjects had their eyes closed, the M/L RMS, EA, and M/L MPF metrics trended larger, and PZ smaller, for both feedback conditions.

MPF analysis for subjects with vestibular deficits showed that provision of vibrotactile feedback significantly increased low frequency power in the 0.1 Hz to 0.4 Hz range for the M/L direction during Romberg stance with eyes open and with eyes closed.

### Subjective evaluation of cell phone based balance trainer by all subjects

According to the post-experimental Likert questionnaire, the majority of subjects (healthy and those with vestibular deficits) agreed that their body was more stable when vibrotactile feedback was available than when it was not (Q1 - avg. 3.95/5), that vibrotactile feedback did not distract them from performing the given balance task (Q2 - avg. 4.21/5), and that they felt more confident in their ability to maintain balance when vibrotactile feedback was available (Q3 - avg. 3.95/5). Furthermore, subjects agreed that they could use the device at home by themselves if it were provided to them as-is (Q4 - avg. 4.21/5) and most subjects understood how to use the vibrotactile feedback provided (Q5 - avg. 4.68/5). Finally, when subjects were asked to rate their preference for *not *using vibrotactile feedback during stance tasks, they disagreed with the statement (Q6 - avg. 2.42/5). The subjects with vestibular deficits consistently rated the utility of the feedback higher than the healthy subjects: Q1 - 4.50 vs. 3.80; Q2 - 4.50 vs. 4.13; Q3 - 4.75 vs. 3.73; Q4 - 4.25 vs. 4.20; Q5 - 5.00 vs. 4.60; Q6 - 1.25 vs. 2.73.

## Discussion

The main finding of this work is that body tilt can be robustly captured by a cell phone equipped with a tri-axial accelerometer and used to assist balance, offering a system that has a number of advantages in terms of cost, size, weight, functionality, flexibility, and accessibility versus more complex laboratory-based and commercial systems dedicated to this purpose. Comparisons of postural tracking performance between the cell phone system and a sophisticated IMU technology often implemented in non-portable lab-based systems [27,35,36,40] demonstrate that the cell phone system can provide postural tracking with resolution better than 0.2°. Moreover, the EKF-based motion estimation algorithm implemented in the cell phone system was shown to largely eliminate inherent sensor noise and provide a robust estimate for body tilt without significant time delay.

Since the cell phone based system studied here only incorporates a tri-axial accelerometer, body tilt estimation is inferior to IMU systems that incorporate both an accelerometer and a gyroscope. This limitation could be overcome by employing additional sensors, such as a tri-axial gyroscope that measures angular velocities or a tri-axial magnetometer that measures the earth's magnetic field. A second concern is that the system studied here requires the cell phone to be placed on the body segment being evaluated, since the sensor capturing body motion is embedded within the phone itself. Consequently, we are currently developing a second-generation cell phone system that incorporates a small IMU (containing both a tri-axial accelerometer and a gyroscope). Finally, since the system was tested using only a small number of subjects, some of the true differences between conditions may not have been detected in the analysis.

The results of this proof-of-concept study show that healthy subjects exhibit significant improvements in the most challenging stance condition (tandem Romberg) when cell phone based vibrotactile feedback is provided. Indeed, the majority of healthy subjects remained inside the given dead zone for the stance tasks executed.

The MPF results suggest that subjects make more frequent corrections of tilt in the presence of vibrotactile feedback in order to remain in the dead zone, contributing to an increase in mean power frequency; i.e., they actively move their trunk in response to the cues as opposed to stiffening their bodies and remaining as still as possible in order to prevent movement outside of the dead zone. In the case of M/L MPF, however, results for tandem Romberg stance in the presence of vibrotactile feedback showed a non-significant increase in comparison to the feedback-off trials. This can be explained by the challenging nature of this stance, especially when performed with the eyes closed; in these trials, subjects may have stiffened their bodies in order to remain still, and did not search for and use the limits of stability coded into the vibrotactile feedback. Consequently, sway frequency differences between the feedback-on and feedback-off testing conditions were insignificant.

Similar findings were observed in subjects with vestibular deficits, except for the EA metric, for which subjects showed an insignificant decrease during eyes-open trials. Furthermore, for this group the main effects of vibrotactile feedback were observed only in the direction in which vibrotactile feedback was provided. For example, neither A/P RMS nor A/P MPF showed significant changes in the presence or absence of vibrotactile feedback in either eyes-open or eyes-closed conditions when feedback was provided solely in the M/L direction. Performance metrics in the directions without vibrotactile feedback could be improved by providing additional tactors; we have previously shown that a four-tactor vibrotactile feedback paradigm can be used by subjects with vestibular loss to reduce RMS body sway [27,30,39]. These findings prompted the next-generation cell phone system to be designed such that it supports up to four tactors; however, evaluation of the effectiveness of a four-tactor configuration was not conducted in this study.

The collected survey results indicate that the majority of subjects feel that the proposed cell phone balance training system could be used at home without difficulty. Subjects with vestibular deficits indicated a higher confidence level (avg. 4.75/5) than healthy subjects (avg. 3.73/5) in their ability to maintain balance with the aid of vibrotactile feedback. This is consistent with the improved balance metric outcomes measured in subjects with vestibular deficits when feedback was present, suggesting that these individuals can adequately rely on the supplemental body motion cues provided by vibrotactile feedback.

## Conclusion

This paper describes the design, development, and assessment of a cell phone based vibrotactile feedback system intended for balance rehabilitation training. Based on the experimental study and survey evaluation conducted with this system in healthy subjects and those with vestibular deficits, we demonstrated that the prototype can be used to provide real-time feedback during a subset of balance rehabilitation exercises. In addition, due to the system's ability to store motion data, the effects of assistive feedback can be quantified through subsequent analysis.

This work provides proof of concept for a portable balance rehabilitation system that has potential for broad accessibility. To the best of the authors' knowledge, this is the first implementation of vibrotactile instructional feedback in a portable cell phone platform as a means to improve balance.

## List of abbreviations

ANOVA: analysis of variance; A/P: anterior-posterior; EA: elliptical area; EKF: extended Kalman filter; LMM: linear mixed effects models; MCU: microcontroller unit; M/L: medial-lateral; MPF: mean power frequency; RMS: root mean square; PZ: percent time within dead zone; SDK: software development kit.

## Competing interests

The authors declare that they have no competing interests.

## Authors' contributions

B-CL developed and implemented the tilt estimation algorithms, assisted with software development, participated in the design of the study, collected the data for healthy subjects and subjects with vestibular deficits, and helped to draft the manuscript. JK developed the initial prototype. SC, a researcher at the Claude Pepper Center Biostatistics Core, conducted the statistical analysis. KS conceived of the cell phone based vibrotactile feedback system and study, supervised the research, and helped to draft the manuscript. All authors read and approved the final manuscript.
